# Visualizing the morphology of vortex lattice domains in a bulk type-II superconductor

**DOI:** 10.1038/ncomms9813

**Published:** 2015-11-02

**Authors:** T. Reimann, S. Mühlbauer, M. Schulz, B. Betz, A. Kaestner, V. Pipich, P. Böni, C. Grünzweig

**Affiliations:** 1 Heinz Maier-Leibnitz Zentrum (MLZ), Technische Universität München, D-85748 Garching, Germany; 2 Physik-Department E21, Technische Universität München, D-85748 Garching, Germany; 3 Paul Scherrer Institut, Neutron Imaging and Activation Group, CH-5232 Villigen, Switzerland; 4 Jülich Centre for Neutron Science (JCNS) at Heinz Maier-Leibnitz Zentrum (MLZ), Forschungszentrum Jülich GmbH, D-85748 Garching, Germany

## Abstract

Alike materials in the solid state, the phase diagram of type-II superconductors exhibit crystalline, amorphous, liquid and spatially inhomogeneous phases. The multitude of different phases of vortex matter has thence proven to act as almost ideal model system for the study of both the underlying properties of superconductivity but also of general phenomena such as domain nucleation and morphology. Here we show how neutron grating interferometry yields detailed information on the vortex lattice and its domain structure in the intermediate mixed state of a type-II niobium superconductor. In particular, we identify the nucleation regions, how the intermediate mixed state expands, and where it finally evolves into the Shubnikov phase. Moreover, we complement the results obtained from neutron grating interferometry by small-angle neutron scattering that confirm the spatially resolved morphology found in the intermediate mixed state, and very small-angle neutron scattering that confirm the domain structure of the vortex lattice.

## Abstract (web summary)

The phase diagram of type-II superconductors exhibits a multitude of different phases, whose study can shed light on domain nucleation and morphology. Here the authors use neutron grating interferometry to investigate the nucleation and phase changes of an intermediate mixed state in a niobium superconductor.

## Graphical abstract

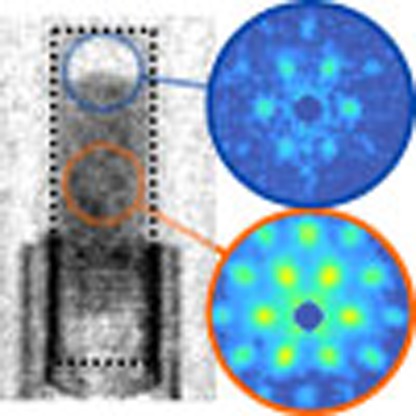

Domain structures with striking similarities can be found in physical, biological and chemical systems with varying length scales, being abundant on the micrometre scale^1^. Although the physical effects causing the domain patterns in these systems may be different, the reason for domain nucleation is mostly a competition between interactions favouring uniform phase distributions and interactions preferring a phase separation into small unequal domains. As a consequence, all these systems feature similar domain morphologies including stripe, bubble or dendritic patterns. Their theoretical treatment is mostly based on the universal idea developed 80 years ago by Landau and Lifschitz^2^. In this model, domain nucleation originates from an energy minimization of interfacial (surface) energy and nonlocal interactions as for example, demagnetizing fields. A common feature of domain patterns is a strong dependence on the sample history caused by barriers for domain nucleation, impurities, or anisotropies in the underlying interactions^3^,^4^,^5^. However, the influence of both microscopic and macroscopic effects on the domain morphology is not well understood^6^.

Niobium (Nb) type-II bulk superconductors, have proven to act as an almost ideal, clean model system to investigate domain formation as well as for systematic studies of vortex matter^7^,^8^,^9^. Generally, the vortex–vortex interaction of type-II superconductors in the Shubnikov phase is repulsive leading to the well-known Abrikosov vortex lattice (VL)^10^. However, for materials with a low Ginzburg–Landau parameter *κ* such as niobium, a sizeable short range attractive interaction is present in addition to the long-range repulsion^11^,^12^. As a consequence, the transition from the Meissner to the Shubnikov phase at the lower critical field (*H*_C1_) is first order and is accompanied by a discontinuity in the intervortex lattice spacing *d*_VL_. In samples with a non-zero demagnetization coefficient *N*, an intermediate mixed state (IMS) phase is formed in which isolated Shubnikov domains with a typical diameter *d*_IMS_ are nucleated, surrounded by the field-free Meissner state. Figure 1 shows a generic *B*-*T* phase diagram of Nb as well as a schematic depiction of the IMS and Shubnikov phase including their characteristic length scales *d*_VL_ and *d*_IMS_, respectively.

In analogy to the intermediate state of a type-I superconductor, the IMS features all the aforementioned universal domain shapes as was first observed by surface decoration techniques more than 40 years ago^13^. The morphology of the IMS domains is mainly governed by geometric barriers (topological hysteresis) preventing domain nucleation^5^, surface barriers which hamper the entrance of flux lines into the sample^3^,^14^, VL anisotropies^7^ as well as pinning forces^15^. Superconducting vortex matter hence offers the unique possibility to study both phenomena of general importance such as domain nucleation and topology^16^ but also the microscopic origins of superconductivity such as anisotropies in the order parameter^7^. Moreover, in contrast to domains in other systems the vortex density, the vortex interaction and the volume filling of the sample can be ideally tuned by varying magnetic field and temperature.

The real-space visualization of vortex matter is limited to surface-sensitive methods such as Bitter decoration^17^, scanning tunnelling microscopy^18^ and magneto-optical imaging^19^. In contrast, Lorentz-microscopy^20^ and electron holography^21^ are typically used to investigate thin-film samples. Until now, domain structures of the intermediate state^22^,^23^,^24^,^25^ and IMS^12^,^13^,^26^ could only be observed with the help of surface-sensitive techniques.

In contrast, neutrons can easily penetrate bulk samples and interact with the local magnetic field distributions due to their magnetic moment. For instance, the elementary ‘crystallographic' properties of vortex lattices, for example, their symmetry or elasticity are routinely probed by means of small-angle neutron scattering (SANS)^7^,^8^,^9^,^27^. However, as SANS is an integral scattering method, only scattering patterns averaged over the whole sample are recorded. This leaves inhomogeneous VL distributions unresolved, which are present in non-ellipsoidal samples due to geometrical constraints on the penetrating vortex lines^14^,^28^. Moreover, because the typical length scales in the IMS phase are of the order of several micrometres, only indirect evidence of the IMS can be found by SANS^7^,^29^,^30^,^31^ leading to a lack of information on the IMS morphology in bulk samples.

Here we provide an experimental approach for a comprehensive study of the properties of domain nucleation in the IMS phase of a high-purity type-II superconducting niobium rod, particularly a bulk sample with non-ellipsoidal geometry. In previous SANS studies^7^ on the same niobium sample, the IMS phase was only indirectly observed by its hallmark; a constant VL spacing for intermediate magnetic fields^30^. For our investigation we combine three neutron techniques: neutron grating interferometry (nGI) for imaging direct space, and two scattering methods: small-angle scattering and very small-angle scattering (VSANS), which provide complementary information in the reciprocal space. With nGI, being sensitive to micrometre length scales, we obtain spatially resolved maps of the IMS bulk domain distribution, hence, local information. With SANS being sensitive to length scales between 10 and 200 nm we probe the VL within the individual vortex matter domains without any information about the distribution of the domains in the IMS phase. Finally, with VSANS being sensitive to length scales up to 10 μm, we provide statistical information about the distribution of the characteristic sizes of the IMS domains. We directly show that in the case of a bulk non-ellipsoidal, pinning free superconducting specimen, the IMS phase nucleates in the center of the sample and its distribution is strongly inhomogeneous. We then discuss our experimental findings with a particular focus on the impact of our unified experimental approach for studying domain formation in general.

## Results

### Experimental conditions

**Figure 1:**
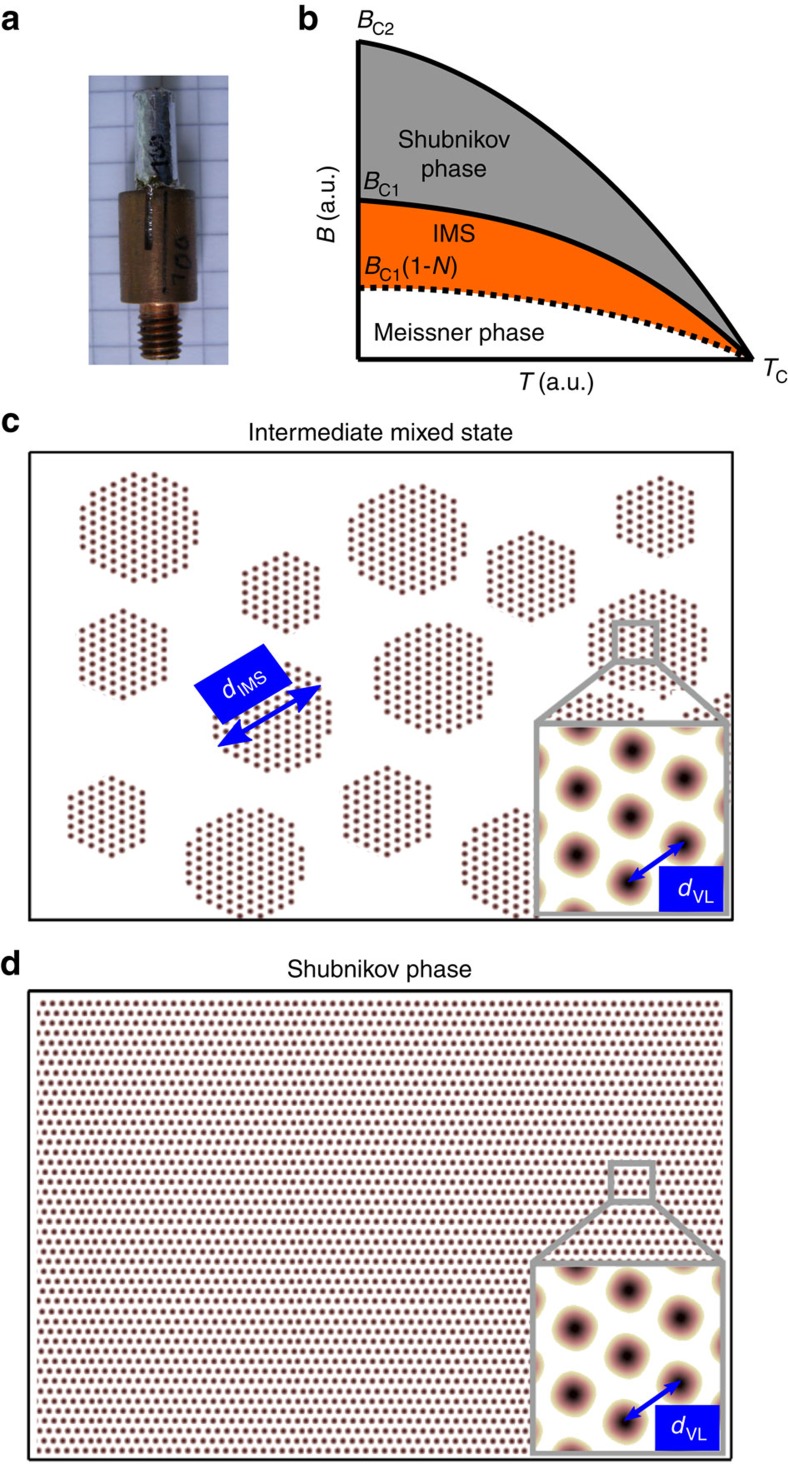
Schematic *B*-*T* phase diagram of the niobium rod. (**a**) Image of the Nb rod investigated in this study. (**b**) Generic phase diagram of the sample: Because of the non-zero demagnetization coefficient *N* of the Nb rod an IMS phase emerges in fields *B*_C1_(1-*N*)≤*B*≤*B*_C1_. The corresponding schematic drawings of the (**c**) IMS phase and (**d**) Shubnikov phase show the characteristic length scales of the intervortex lattice distance *d*_VL_ and IMS domain size *d*_IMS_.

For the experimental study of the IMS phase, we selected a cylindrical rod of ultrahigh-purity niobium (*κ*=*λ/ξ*≈0.74) with a length of 20 mm and a diameter of 4.5 mm (Fig. 1). Niobium is a classical phonon mediated type-II superconductor with a *T*_C_ of 9.2 K. The residual resistivity ratio was measured with an eddy current decay method and was found to exceed ∼10^4^, underlining the exceptional crystallographic quality. In previous neutron scattering experiments^7^,^8^ no signs of volume pinning and trapped flux have been found. The cylinder axis of the sample corresponds to a crystalline [110] direction. For all experiments the magnetic field was applied in the (110) plane, parallel to the incident neutron beam and perpendicular to the cylinder axis. The demagnetizing factor was calculated to be *N*≈0.47. To guarantee a reproducible VL state, all data for different magnetic fields were taken after zero-field cooling (ZFC) deep inside the superconducting phase at *T*=4 K. To exclude effects arising from slightly different magnetic field or temperature conditions, the same sample environment was used for the nGI, SANS and the VSANS measurements.

### Small-angle neutron scattering

**Figure 2:**
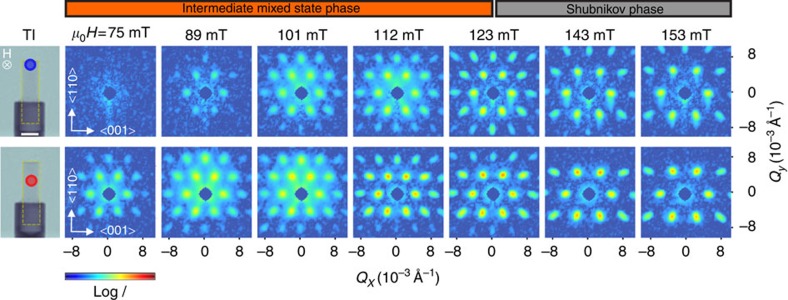
Local SANS patterns of an ultrahigh-purity Nb rod versus magnetic field. The data were obtained after ZFC to 4 K. Measurement positions at the upper edge (top row) and in the centre (bottom row) of the sample were selected using a 3 mm Cd aperture as shown in the TI images on the left side (compare Fig. 5). Scale bar, 5 mm.

To locally probe the crystallographic properties of the VL, SANS experiments are performed at the SANS-1 instrument operated by TUM and HZG at the Heinz Maier–Leibnitz Zentrum (MLZ) Garching^32^. Figure 2 shows typical SANS patterns measured in magnetic fields 75 mT≤*μ*_0_*H*≤153 mT after ZFC to 4 K. Each data set corresponds to a sum over a rocking scan with respect to the vertical sample axis. The data were corrected for background using the zero-field pattern. Using a small aperture mask of only 3 mm in diameter, two different sample positions were probed as indicated in Fig. 2. The data set originating from the position close to the upper sample edge (blue marker) is shown in the top row, the other set from the centre of the sample (red marker) is shown in the bottom row in Fig. 2, respectively. The six-fold symmetry of the VL along this field direction agrees with literature^7^.

The direct comparison of the two measurement positions reveals that the scattering patterns of the VL strongly depend on the position. A clear sixfold pattern is observed in the middle of the sample already at 75 mT. In contrast there is no sign of a VL from the SANS pattern determined at the edge of the rod until the field reaches a value of 89 mT.

**Figure 3:**
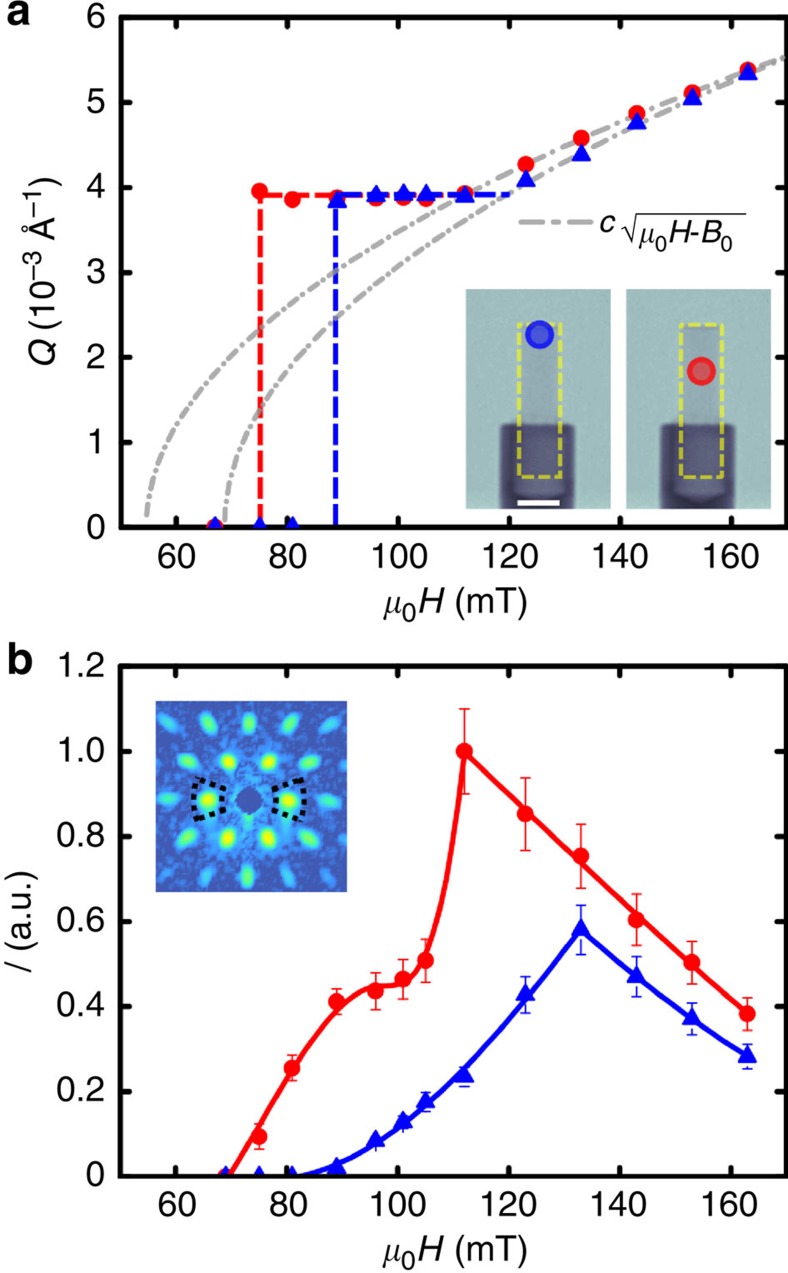
Quantitative analysis of the SANS data from the Nb rod. The two measurement positions are indicated in the TIs in inset of (**a**). Scale bar, 5 mm. (**a**) *Q*-position of the first order Bragg peak of the VL as function of the applied magnetic field. The grey line indicates the expected field dependence (*μ*_0_*H*−*B*_0_)^0.5^ at high fields. The dashed lines are a guide to the eye of the constant Q behaviour in the IMS. (**b**) Integrated intensity of the first order Bragg peak versus magnetic field. The error bars corresponds to the standard error of the mean (s.e.m.). The lines are guide to the eyes. The evaluated Bragg peaks are exemplarily shown in the inset of **b** for *μ*_0_*H*=153 mT.

The *Q*-spacing *Q=2π/d*_VL_ extracted from the SANS data as a function of the applied magnetic field is presented in Fig. 3. The hallmark of the IMS phase, a constant spacing of the VL^30^ corresponding to *Q*_0_=3.9 × 10^−3^ Å^−1^ is obvious for both positions as seen in Fig. 3a. The data reveal that the spacing of the vortex lattice in the IMS is independent of the position of the sample. However, the field regions of the IMS phase differ: For the centre position, the VL domain nucleation sets in at 75 mT and persists up to 112 mT. At the edge position, the VL appears first at 89 mT and is constant until *μ*_0_*H* reaches 123 mT. In higher fields both data sets follow the well-known (*μ*_0_*H*−*B*_0_)^1/2^ (ref. 30) behaviour expected for the Shubnikov phase. The parameter *B*_*0*_ reveals a difference of 14 mT for the two positions.

We emphasize that the delay in appearance of the VL at the top of the sample cannot be explained by an inhomogeneity of the magnetic field. After a careful examination of different rocking angles, a missing scattering signal at the edges due to a bending of the vortex lines can be ruled out as well.

Figure 3b shows the integrated intensity of the first order Bragg peaks as extracted from rocking scans for both positions indicated in the inset. The well-defined maximum marks the transition from the IMS-phase to the Shubnikov phase at *H*_C1_. Again, the shift in magnetic field in between the two positions is clearly visible. The change from the constant *Q*-regime to the smooth high-field behaviour is a second indication for the transition, which is found between 105 and 112 mT for the middle position and between 123 and 133 mT for the upper edge of the sample. The *I*(*μ*_0_*H*) trend as well as *Q*_0_ is in agreement with previous measurements^7^.

However, a closer inspection of the intensity curve of the middle position reveals a nonlinear behaviour with a downward dip at 90 mT. This dip was not observed in former experiments where the entire volume of the sample was probed using a larger aperture. If the intensities of the top and centre positions are added, then the dip is washed out leading to close agreement with literature^7^. Altogether with the delayed appearance of the VL at the edge position of the sample this indicates a strongly inhomogeneous VL domain distribution within the IMS in non-ellipsoidal samples.

### Neutron grating interferometry

Neutron grating interferometry is an advanced neutron-imaging method that is able to locally mark micrometre inhomogeneities due to the distortion of the neutron wave front caused by scattering at ultra small angles. It was previously shown that nGI can be used for phase contrast imaging^33^, dark-field imaging^34^ and tomography^35^ as well as for magnetic domain imaging^36^,^37^,^38^,^39^. In our work nGI is used to directly visualize the inhomogeneous VL domain formation indicated by SANS resulting in a spatially resolved scattering map of the IMS domain distribution.

**Figure 4:**
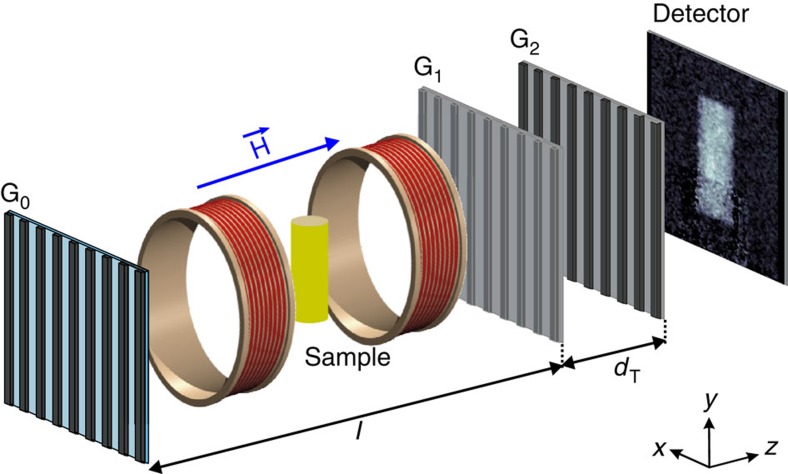
Experimental set-up. Schematic depiction of the set-up of the grating interferometer used for the investigation of the domain formation of the vortex lattice in Nb. The set-up consists of the source grating G_0_, the phase grating G_1_ at distance *l*, the analyser grating G_2_ at distance *d*_T_ behind G_1_, an imaging detector, and the cryomagnetic sample environment (yellow cylinder).

The neutron grating interferometry experiments were carried out at the Paul Scherrer Institut at the Swiss Spallation Neutron Source (SINQ) using the cold neutron imaging facility ICON^40^. The nGI beam line involves the installation of a set of three diffraction gratings at a classical neutron imaging beam line^41^. A schematic depiction of the grating set-up including the cryomagnetic sample environment is shown in Fig. 4.

**Figure 5:**
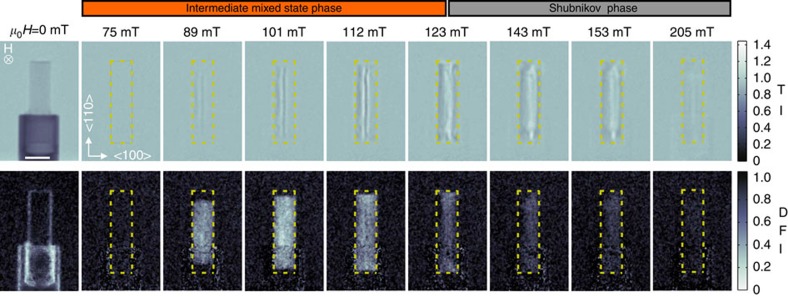
Transmission and dark-field images of an ultra-pure niobium rod as a function of magnetic field. The magnetic field was successively increased after ZFC to *T*=4 K. The contour of the sample is indicated by the yellow-dashed boxes. Scale bar, 5 mm. The TI (top row) and DFI (bottom row) results for *B*>0 are normalized with the results at *μ*_0_*H*=0 mT, hence only the pure magnetic contribution from the VL is visualized. The TI and the DFI provide information about the flux line lattice within the vortex domains and the VL domain formation in the IMS phase, respectively.

The results of the nGI experiments are presented in Fig. 5. The nGI data are grouped in transmission images (TI) in the top row and dark-field images (DFI) in the bottom row. The TI provides information about the local structure of the VL. In contrast to integral scattering techniques such as SANS, the TI locally shows, where the angle enclosed by the incoming neutrons and the orientation of the VL fulfils the Bragg condition. Hence, neutrons that are locally scattered away from their original direction lead to a decrease of the intensity at the corresponding position on the detector (TI<1) and an increase of the general background of diffuse scattering (Supplementary Note 1). The DFI provides information on the domain formation of the VL namely the IMS phase. The signal is caused by neutrons losing their coherence due to ultra small-angle scattering at the IMS domains with length scales in the micrometre range. The sensitivity of the DFI to different sized structures is specified in more detail in the Supplementary Note 3 and a calculated sensitivity curve for the used nGI set-up is shown in Supplementary Fig. 2.

At 0 mT, the contrast seen in the TI is caused by the attenuation of the beam by the sample and its holder (compare inset of Fig. 1). In the DFI, residual background scattering from the sample holder and the edges of the sample is seen. All TIs and DFIs for finite magnetic fields were normalized to the data for 0 mT, hence the shown TI and DFI contrasts for *μ*_0_*H*≥75 mT are caused by the appearance of the vortex lattice only and are of purely magnetic origin.

If the magnetic field is increased after ZFC to 4 K, then the sample passes through the different superconducting phases. At 75 mT, the sample enters the IMS phase as a VL is present in the sample as indicated by the SANS experiments from Fig. 2. However, the scattered intensity is too weak to produce sufficient contrast in the TI and DFI. At 89 mT and above, both DFI and TI show a clear contribution caused by the VL and its IMS domains inside the sample. The TIs show a line shaped contrast persisting up to the highest field of 205 mT, with its maximum at 112 mT. This particular contrast variation in the TI is attributed to a slight distortion of vortices in the plane perpendicular to the cylinder axis, which leads to a horizontal variation of Bragg angles within the sample (Supplementary Fig. 1).

A pronounced signal in the DFIs originating from the IMS domains exists for magnetic fields between 89 mT≤*μ*_0_*H*≤123 mT with a maximum at 101 mT. For 89 and 101 mT a homogenous DFI contrast is observed except at the top and bottom ends. At 112 mT a homogenous contrast is formed where also the top and bottom ends contribute to the DFI. The variation of the contrast of the DFI with increasing magnetic field inside the IMS phase is attributed to the increasing filling factor of the sample leading to an enlargement of the domain sizes beyond the sensitivity range of the nGI set-up. At 123 mT the sample is characterized by a phase coexistence of IMS and Shubnikov phase. The interpretation of the TI signal together with DFI and SANS indicates that the bottom and top part are still in the IMS phase causing a DFI signal, whereas in the central part of the sample only a weak indication for a domain structure is detected.

At high magnetic fields *μ*_0_*H*≥143 mT the Shubnikov phase completely fills the sample and contrast is seen in the TI only. Similar to the SANS data, the decreasing intensity observed with increasing field is explained by the field dependent form-factor of the vortices^30^. The remaining contribution in the DFI is attributed to the cross-talk originating from a TI signal greater then unity. A detailed quantitative explanation of the cross-talk is given in the Supplementary Note 4 and its influence on the DFI is visualized in Supplementary Fig. 3. The cross-talk can be avoided by choosing a geometry with the field perpendicular to the beam direction. In this case, the VL is rotated out of the Bragg condition preventing SANS scattering which influences the TI and causes the cross-talk effect. However, the isotropic ultra small-angle scattering signal of the IMS, which is largely invariant under this rotation will still cause a DFI contrast. nGI measurements in this geometry are shown in the Supplementary Fig. 4 and discussed in the Supplementary Note 5. DFI results obtained in the perpendicular field geometry reveal a similar domain distribution as the ones shown in Fig. 5, but lack any information on the morphology of the VL seen in the TI.

Note that it is not yet possible to extract quantitative information on the size distribution of the IMS domains from nGI. However, nGI provides a proof of their existence by matching the sensitive range of the nGI set-up (Supplementary Note 3).

### Very small-angle neutron scattering

To quantify our findings on the IMS domains as obtained by DFI, VSANS experiments have been performed at the KWS-3 beam line operated by Jülich Centre for Neutron Science (JCNS) at the MLZ^42^.

**Figure 6:**
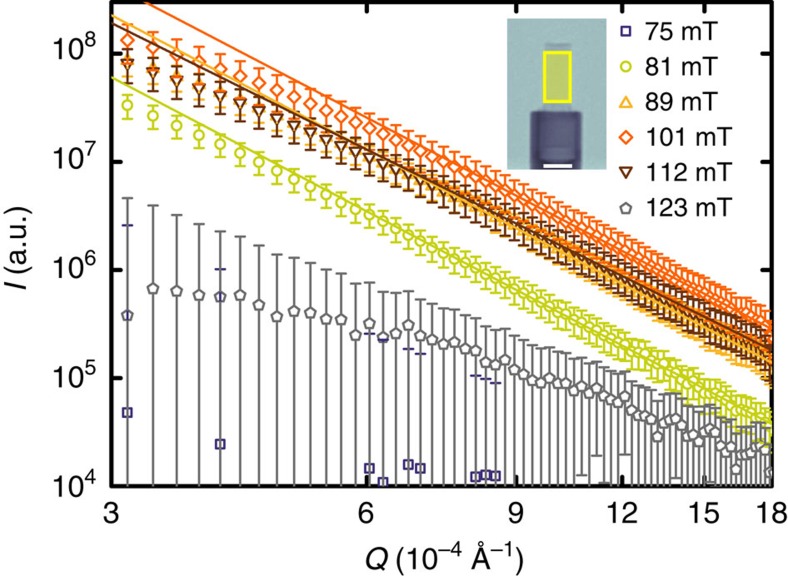
VSANS scattering curves of an ultrahigh-purity Nb rod. The data were obtained after ZFC to 4 K in different magnetic fields. The Cd aperture mask is depicted as the yellow area seen in the inset. Scale bar, 5 mm. Error bars are calculated from the propagation of uncertainty of the sample transmission and the s.e.m. of the scattering curves measured at *B*=0 and in the corresponding field, respectively. The high Q regime can be approximated via a power law behaviour *I*∼
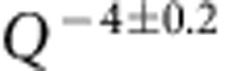
, which is shown as solid lines in the graph.

Figure 6 shows typical VSANS data. Normalized, radially averaged scattering curves are shown as a function of the momentum transfer *Q* for magnetic fields between 75 and 123 mT after ZFC to 4 K. The radial averaging was performed over two 30° sectors. For each magnetic field, the sample transmission was obtained by normalization to the direct beam: The scattering curves were obtained by a normalization of the scattering pattern measured in an applied field to its transmission and subsequent subtraction of the zero-field scattering pattern. Consequently, the data presented in Fig. 6 show only magnetic-scattering contributions.

Likewise in the DFI in Fig. 5, no additional scattering is observed at 75 mT. In the field region between 81 and 112 mT a scattering signal is found, which vanishes in fields above 123 mT. The VSANS signal in intermediate fields is caused by neutrons scattered off the IMS domains and precisely coincides with the DFI contrast seen with nGI. Moreover, as seen in Fig. 6 the scattering curves decreases as a power law *Q*^−α^ for *Q*>6 × 10^−4 ^Å^−1^ in this field range. The exponent α varies between 4.1±0.1 for fields from 81 to 101 mT and 3.9±0.1 at 112 mT. In higher fields, the scattering vanishes again because the sample enters the Shubnikov phase. SANS scattering off the VL still exists but the scattering angles are too large to hit the VSANS detector. The scattering shows up as a correction to the transmission of the sample. The domain structure at the edges of the sample seen with DFI at 123 mT is not detectable by VSANS because it is not covered by a Cd aperture placed in front of the Nb rod as shown in Fig. 6 (yellow box seen in the inset).

The VSANS signal in the IMS phase cannot be explained by neutrons scattered off the vortex lattice itself or by a large distribution of the VL parameter *d*_VL_, as the complementary SANS data reveal well-defined Bragg peaks with high intensity from the vortex lattice at much larger *Q*-values (*Q*≅4·10^−3^ Å^−1^). The high transmission of the sample of 0.9 even in the IMS phase indicates that multiple scattering can be neglected as source of the VSANS signal. The *Q*-resolved VSANS data hence clearly reveals μm sized magnetic-scattering contributions for field values attributed to the IMS via nGI and indirectly by SANS.

In summary, a comparison of the different neutron techniques applied for our study of the VL structure and its IMS domain morphology is shown in Tab. 1. Further detailed information on the techniques, their corresponding reciprocal and direct space sensitivities, their spatial resolution and the contrast mechanism are given in the methods section and the Supplementary Notes 1–3.

## Discussion

A comprehensive interpretation of the VL domain structure of the IMS and its evolution with increasing field based on the combination of nGI, SANS and VSANS data are given in the following paragraphs.

The visualization of the VL domain distribution obtained by means of nGI is in perfect agreement with the SANS and VSANS results. Hence, our study clearly demonstrates the capability of nGI to image the IMS nucleation and to determine the phase boundaries in the superconducting phase of Nb. For the case of IMS nucleation in a cylindrical specimen we show that the IMS domain nucleation starts in the center of the rod and the IMS region propagates along the cylinder axis to the edges, the IMS finally evolves into the Shubnikov phase at the edge of the sample, the induction at the top and bottom part of the sample reveals a delay of 14 mT with respect to the central part, and a coexistence of pure Shubnikov phase and IMS is possible due to demagnetization effects in non-ellipsoidal geometries.

Note that after ZFC the vortices can only penetrate the sample from the outside of the superconducting sample. Therefore, the observed IMS nucleation in the middle is peculiar. Different authors treated the problem of field penetration into type-II superconductors^14^,^28^,^43^ and addressed the influence of sample shape onto the magnetic hysteresis of the superconducting samples. Although the focus of these studies lies on the penetration of VL into discs and slabs aligned perpendicular to the applied field as well as into cylinders and rods in parallel-field configuration some of their main results can be generalized to the presented case of the Nb rod and explain the peculiarities: In the absence of pinning, the Lorentz force pushes the VL to the middle of the sample once the flux lines have penetrated the sample^14^ and overcome the geometrical barrier at the top and bottom edges of the rod. As the VL nucleation in the middle of the sample generates a dipolar field opposed to the applied one, the induction at the edge of the samples is reduced. Hence, the IMS nucleation as well as the IMS to Shubnikov transition is delayed at these positions. We could quantify this delay to 14 mT.

Besides these hand-waving arguments, it has to be pointed out that the process of the IMS nucleation is not well understood. A successful theory for IMS modelling has to include the detailed nature of the vortex–vortex interaction, the disturbance of the applied field by the IMS structure, the surface tension between VL and Meissner domains (similar as in the case of the intermediate state^15^,^44^) and the geometrical constrains in the rod. However, as the presented results can be directly compared with theoretical models and the nGI method provides unique access to the bulk domain distribution in this pinning free, cylindrical sample our study may serve as nucleation point to stimulate further detailed experimental and theoretical work.

Quantitative information obtained by VSANS can serve as further input parameter. In flat samples magneto-optical investigations^12^,^45^ as well as numerical calculations suggest the existence of a preferred thickness-dependent IMS domain size similar to the case of the intermediate state^11^,^46^. However, for our sample it was not possible to extract an exact size distribution of the IMS domains from the VSANS scattering curve. Although a clear magnetic-scattering signal is present between 81 and 112 mT in the *I*(*Q*) data, no sign of a preferred domain length is observed. Domain sizes larger than the VSANS sensitivity or a too broad domain size distribution due to the cylindrical shape of the sample may explain this behaviour. However, the Porod *Q*^−4^ (ref. 47) dependence of the scattering curve at large *Q* indicates that regular shaped domains exist having a smooth surface on a sub-micrometre scale. Furthermore, the high scattered intensity and deviations from the power-law behaviour at small *Q* give a hint, that the domain sizes are slightly larger than the range probed by VSANS.

Finally, some effects arising in our data have not been addressed so far. The upward kink in the *I*(*B*) SANS data set (Fig. 3b) recorded at the middle position of the sample is unexpected. The form factor of the vortex lattice is independent of the field due to a constant magnetic induction *B*_0_ in the IMS. In previous studies it was shown that the integrated intensity of a VL Bragg peak inside the IMS phase therefore only depends on the volume fraction of the VL and increases linearly with the applied magnetic field^30^. The presence of an upward kink of the VL intensity recorded at the center position of the sample reveals that a changing local distribution of IMS domain regions is required in addition to the linear increase of volume fraction to explain this behaviour. Besides serving as information for a modelling of the VL penetration, this result has an important impact on the general interpretation of VL SANS data: our data show, that the geometry of the sample influences the local VL configuration even far away from the sample edges. Hence, both the sample shape as well as its illuminated region should always be carefully considered for the interpretation of SANS data.

In summary, we have presented a systematic approach to study the properties of the VL of the bulk type-II superconductor niobium. The combination of nGI, SANS and VSANS coherently merges the spatial resolution of real-space methods with the quantitative statistical information obtained by reciprocal space techniques. We are able to cover a wide length scale from 10 nm to 10 μm and gather detailed information about the crystallographic properties of VLs and their arrangement in the IMS domains without the restrictions implied by the use of thin-film samples for surface-sensitive methods or odd sample shapes for decoration techniques. Furthermore, our nGI measurements provide a direct visualization of the IMS domain nucleation in bulk samples.

As a main finding of our investigation, we could clearly identify an unconventional domain volume filling of the sample within the bulk IMS phase. Furthermore, we show how neutrons can reveal the IMS phase boundaries in niobium. The different superconducting phases which are detected by nGI are fully consistent with the SANS and VSANS results.

The presented study paves the way for theoretical investigations of the IMS domain distribution and a direct comparison of our experimental results to theory, which would yield further insight into the complex physic of flux penetration into superconductors with non-trivial shape. Moreover, our study also sheds light onto the general problem of domain arrangement under fixed boundary conditions.

Finally, our general approach is by no means limited to superconducting systems. The nGI method can be extended to investigate the rich variety of modulated phases appearing in dense packed, liquid and dilute physical, chemical or biological systems and systems near phase transitions, in particular first order phase transitions, as well as in emerging magnetic systems such as skyrmion lattices^48^. All of them share the macroscopic domain formation on the micrometre scale as a common feature.

## Methods

### Cryomagnetic sample environment

A closed cycle cold head cryostat (Sumitomo SHI-RDK-2025D) providing a base temperature of ∼3.7 K was used. The cryostat containing the sample was placed in a normal conducting, water cooled magnet in Helmholtz geometry. The inhomogeneity of the field was measured to be smaller than ±1.0% over the sample volume.

### Small-angle neutron scattering:

A wavelength *λ*=11.9 Å and a sample to detector distance of 20 m was chosen. A Cd aperture mask with a small diameter of 3 mm was placed in front of the sample position after a collimation distance of 20 m. In previous studies, the aperture was chosen such that most of the sample was illuminated. Hence, an integral signal over the whole sample was obtained. The use of the 3-mm aperture permits us to perform locally resolved SANS investigations. The position of the neutron spot on the sample could be determined with an accuracy better than 1 mm.

### Neutron grating interferometry

The source grating G_0_ (periodicity *p*_0_=1,076 μm) is a Gadolinium aperture mask with transmitting slits, placed in a monochromatic beam of neutrons having a wavelength *λ*=4.1 Å and a wavelength spread of Δ*λ*/*λ*=15% provided by a velocity selector. G_0_ creates an array of periodically repeating coherent line sources and effectively allows the use of relatively large, centimeter-sized neutron beams without compromising the coherence requirements for the interferometer formed by G_1_ and G_2_. The silicon phase grating G_1_ (*p*_1_=7.97 μm), placed at a distance *l*=5.23 m behind G_0_ acts as a phase mask and imprints periodic phase modulations onto the incoming wave field. Due to the Talbot effect, this phase modulation is transferred into an intensity modulation at the detector position, where the interference pattern is analysed using the analyser grating G_2_ (*p*_2_=4 μm) made of Gadolinium. Neutrons which are scattered along the path within the interferometer destroy the interference fringes and locally mark the position of the scattering sites as contrast in the DFI. Therefore, the nGI method uses reciprocal space scattering signatures to generate visible contrast in real-space. Details of the nGI data processing for the TI and DFI can be found in the Supplementary Note 2.

The nGI set-up was combined with a state of the art neutron-imaging detection system. The images were recorded using a 100-μm thick ^6^Li/ZnS scintillator screen and a cooled charge-coupled device (CCD; Andor Neo sCMOS, 2560 × 2160 pixels, pixel size: 6.5 μm). The effective spatial resolution of the camera set-up of 100 μm was determined by the intrinsic blurring of the scintillation screen^49^. The rather large dimensions of the cryomagnetic sample environment comes along with a untypical large sample to detector distance of 30 cm for imaging set-ups leading to an effective image resolution of about 0.5 mm.

### Very small-angle neutron scattering

The instrument uses a toroidal focusing mirror to expand the *Q*-resolution down to the 10^−4^ Å^−1^ regime while maintaining a reasonable intensity. The corresponding length scales cannot be probed by conventional pinhole SANS, however, they strongly overlap with the values obtained with nGI. With a sample to detector distance of 8.5 m and a neutron wavelength of *λ*=12.8 Å the instrument was sensitive to length scales up to ∼3 μm and therefore capable to resolve scattering originating from the IMS domains.

## Additional information

**How to cite this article:** Reimann, T. *et al.* Visualizing the morphology of vortex lattice domains in a bulk type-II superconductor. *Nat. Commun.* 6:8813 doi: 10.1038/ncomms9813 (2015).

## Supplementary Material

Supplementary Information Supplementary Figures 1-4, Supplementary Notes 1-5 and Supplementary References

## Figures and Tables

**Table 1:** Comparison of the used techniques for the investigation of the VL structure and IMS domain formation.

**Technique**	**Instrument**	***q*****-range sensitivity (Å^−1^)**	***d*****-range sensitivity (nm)**	**Spatial resolution (determined by)**	**Information about**
SANS	SANS 1	2 × 10^−2^ to 1 × 10^−3^	30–600	3 mm diameter (aperture)	VL
VSANS	KWS 3	3 × 10^−3^ to 1 × 10^−4^	200–6,000	5 mm × 10 mm (aperture)	IMS domains
nGI (TI)	ICON	2 × 10^−1^ to 3 × 10^−3^	3–200	0.5 mm (geom. blurring)	VL
nGI (DFI)	ICON	6 × 10^−3^ to 1 × 10^−5^	100–60,000	0.5 mm (geom. blurring)	IMS domains

DFI, dark-field images; nGI, neutron grating interferometry; SANS, small-angle neutron scattering; TI, transmission images; VSANS, very small-angle neutron scattering.

The *q*-range of the TI was calculated assuming that the scattering angle must be larger than the geometrical blurring caused by the high sample to detector distance (SDD) but smaller than the angular range which is covered by the detector.
