# Supplementary material for: Visualizing the morphology of vortex lattice domains in a bulk type-II superconductor
Source: Nat Commun. 2015 Nov 2;6:8813. doi: 10.1038/ncomms9813 (PMC4667613; doi:10.1038/ncomms9813)
Supplement: Supplementary Information — Supplementary Figures 1-4, Supplementary Notes 1-5 and Supplementary References [file ncomms9813-s1.pdf]

SUPPLEMENTARY INFORMATION

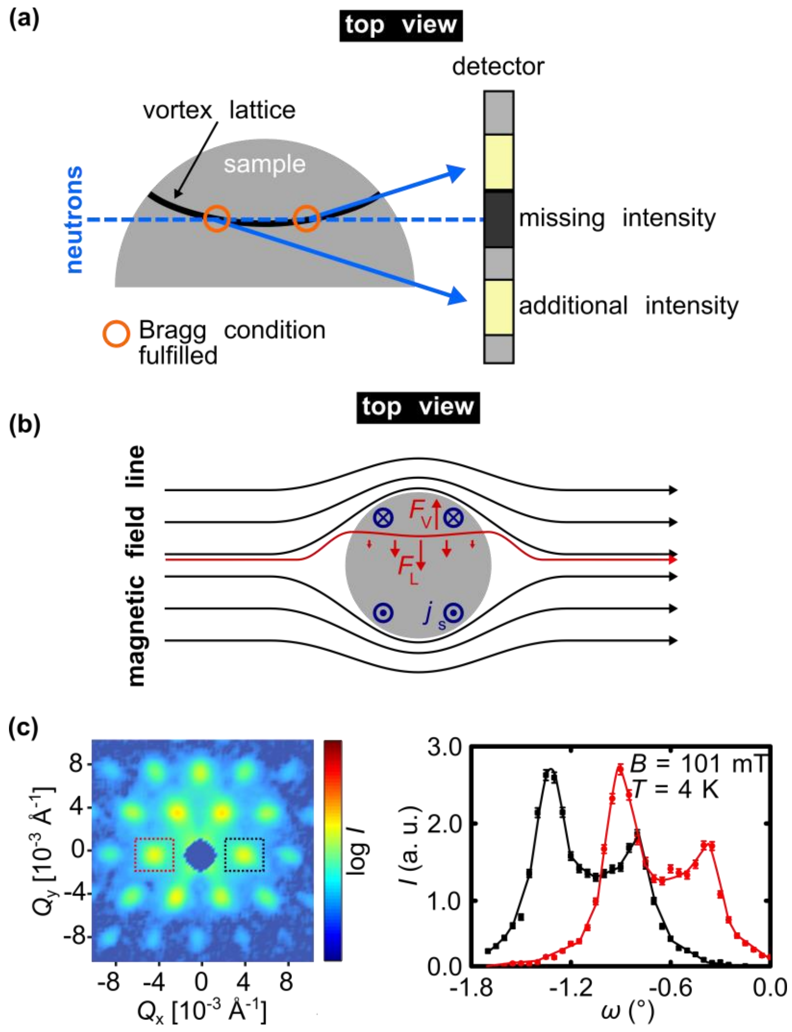

Supplementary Figure 1: On the origin of the TI contrast. a: Schematical sketch, explaining how the curvature of the VL generates a line-shaped contrast variation in the TI. b: Top view sketch of the forces acting on a vortex line upon field penetration. c: SANS Detector pattern and rocking curve of the Nb rod in an applied field of 101 mT after ZFC to 4 K. The measurement positon corresponds to the bottom row of Fig. 2. The double peak structure suggests a symmetrical bending of the flux lines.

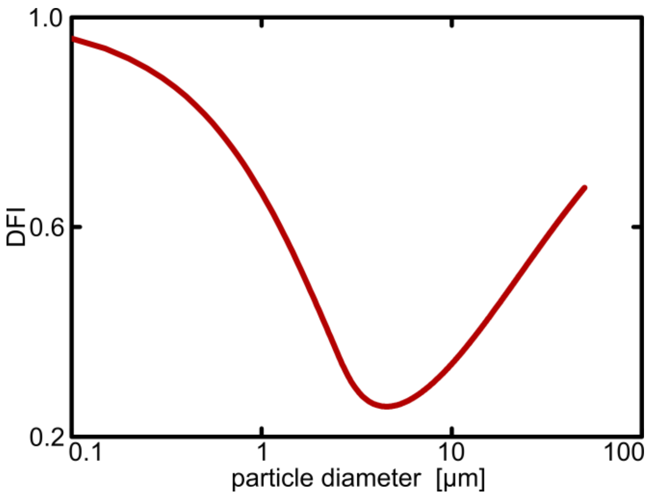

Supplementary Figure 2: DFI reference curve for a 10 % suspension of polystyrene microspheres versus the particle diameter. The plot was calculated using the formula given in [1]. A maximal sensitivity in the range of 1-50 µm is obtained.

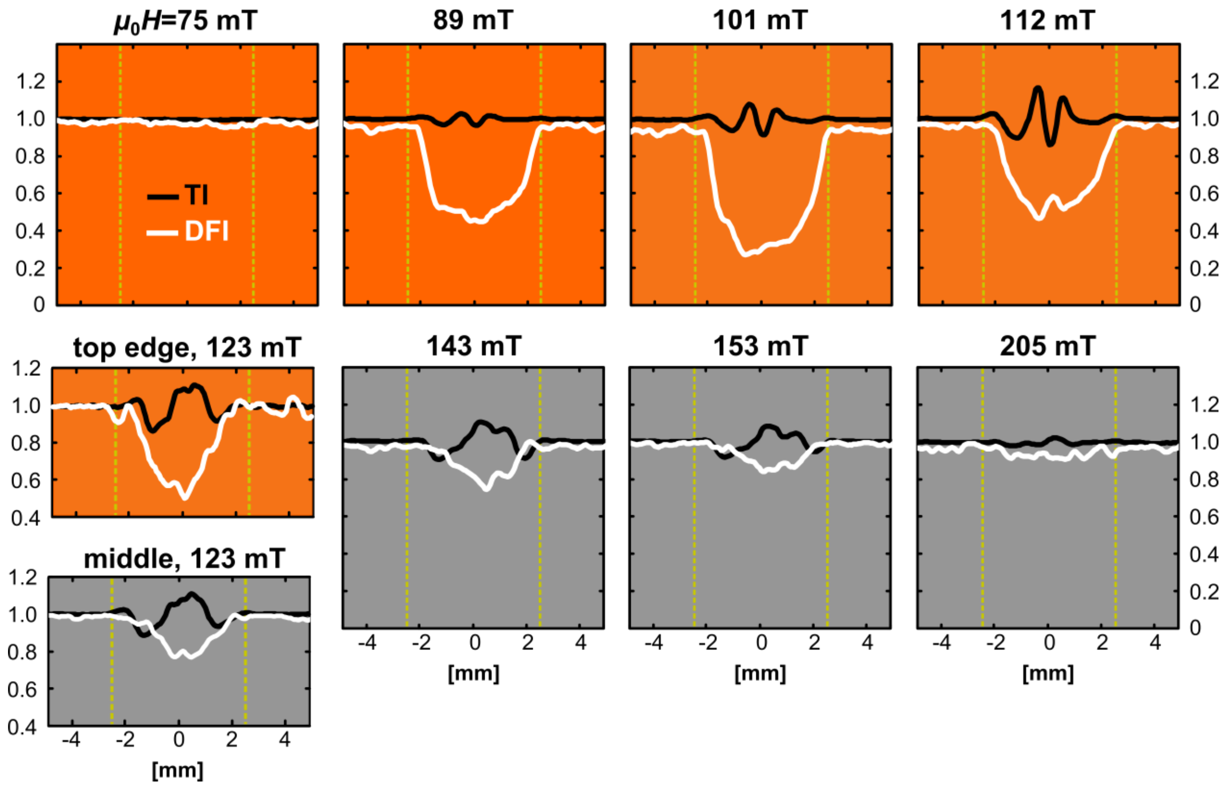

Supplementary Figure 3: Profiles of the TI and DFI signals of the nGI data from Fig. 5. The TI (black) and DFI (white) profiles were measured along a horizontal section in the centre of the rod. The yellow dashed vertical lines indicate the boundary of the sample. Profiles measured along the top edge of the Nb rod are additionally shown for 123 mT. They illustrate the delayed IMS to Shubnikov transition at the sample edge compared to the center of the sample.

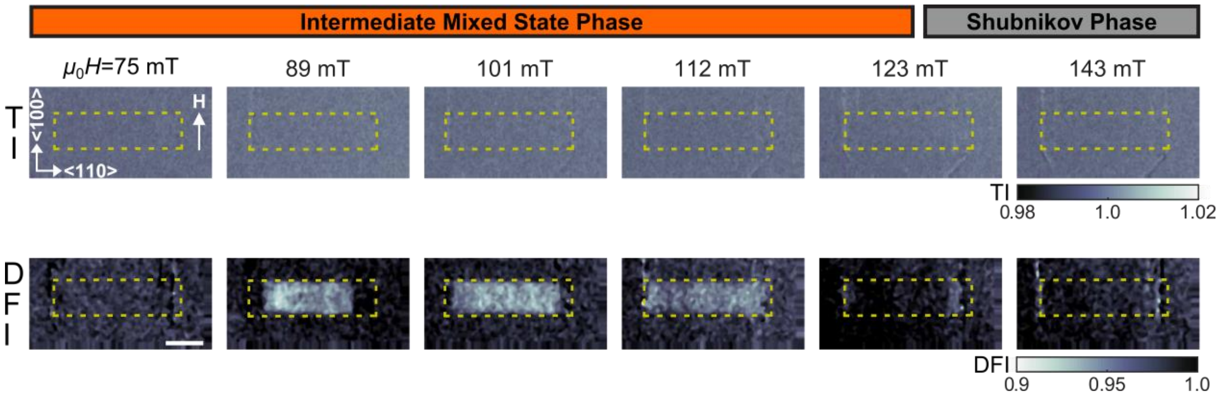

Supplementary Figure 4: TI and DFI results of an ultra-pure Niobium rod as a function of increasing magnetic field after ZFC to *T* = 4 K. The field was applied perpendicular to the neutron beam. The contour of the sample is indicated by the yellow dashed boxes. The TI (top row) and DFI (bottom row) results are normalized with the results with $\mu_0 H$ = 0 mT, hence only the pure magnetic contribution from the VL is visualized. As the VL is rotated away from the Bragg condition no TI contrast arises. However, USANS scattering at the IMS domains, still causes a pronounced DFI contrast. The scale bar corresponds to 5 mm.

Supplementary Note 1: On the origin of the contrast variation in the transmission image

In the following paragraphs we explain how a slight bending of the VL due to demagnetization effects and Lorentz forces acting on the vortices leads to the line-shaped contrast which is observed in the transmission image (TI) presented in the top row of Figure 5.

The TI data of the Nb rod shown in Fig. 5 were taken in different magnetic fields between 0 and 205 mT after ZFC to 4 K. They reveal the emergence of a vertically aligned, line-shaped contrast variation in magnetic fields above 89 mT which slightly changes its morphology in fields higher than 123 mT.

The TI for fields above 0 mT were normalized to the data for 0 mT. Hence, the observed TI contrast variation is of magnetic origin only. It could either be generated by USANS scattering off the domain structure of the IMS (typical domain size of approx. 10 µm) or by SANS scattering off the VL within the IMS domains (lattice spacing 160 nm). For the employed SDD of 300 mm, USANS and SANS scattering on the length scales mentioned above lead to a neutron deflection at the detector position of 0.01 mm and 0.77 mm, respectively. A possible USANS scattering contribution of 0.01mm resulting from the IMS domain structure lies much below the detector resolution of 0.5 mm and hence cannot be resolved. This proves that the TI contrast originates solely from SANS scattering off the VL.

However, SANS scattering will only occur if the angle enclosed by the incoming neutron beam and the orientation of the VL fulfils the Bragg condition. In this case, neutrons which are locally deflected away

from their original direction will lead to a reduced intensity (TI <1) at the corresponding detector position. As direct consequence, some parts of the detector will measure a higher intensity (TI>1) due to the additional number of neutrons scattered to these positions (see Supplementary Figure 1 a).

For the case of a VL, strictly parallel aligned to the external magnetic field direction over the entire sample volume, a TI signal different than unity would only arise if the VL is rotated to the Bragg angle. In this case, the TI would decrease at one sample edge and increase at the other as the intensity is just slightly shifted in the horizontal plane. This is obviously not the case for the TI of the Nb rod presented in Fig. 5. To generate the distinct TI contrast variations of high and low intensity streaks seen between 89 and 112 mT, a spatial variation of the VL orientation (mosaic) is required in addition.

The TI pattern can be concisely explained introducing a symmetrical bending of the VL in the Nb rod within the horizontal plane, as schematically indicated in Supplementary Figure 1 a. In this case neutrons are Bragg scattered twice inside the sample. This leads to the observed arrangement of high and low intensity streaks separated by twice the Bragg angle which corresponds to a stripe distance of 1.5 mm at the detector in accordance to presented TI data. For this effect to occur a bending in the range of the VL Bragg angle (~0.1°) is sufficient.

The existence of a small curvature of the VL can be understood by simplified arguments based on the sample geometry and the balance of forces acting on a flux line penetrating a SC sample (Supplementary Figure 1 b).

Due to the cylindrical geometry of the sample, magnetic flux lines can enter the sample in the horizontal plane without being hampered by a geometrical barrier at the edge of the sample. However, a further penetration of the flux line into the bulk of the sample requires an elongation of the vortex lines and hence a loss of condensation energy, which results in an outward directed force F_V. On the other hand, the flux line is exposed to Meissner screening currents j_s flowing in vertical planes perpendicular to the applied field. As a consequence, Lorentz forces F_L directed to the center of the sample act on the vortex lines. These forces are proportional to the local screening current density which is maximal in the vertical mirror plane of the rod perpendicular to the applied field. Hence, they do not act homogeneously on the vortex line but their magnitude increases towards the centre of the sample which gives rise to the observed flux line distortion.

The bending of the VL can be directly confirmed by a SANS rocking scan in which the intensity of the first order VL Bragg peaks is measured as a function of the vertical rotation angle ω of the sample and the magnetic field. Hence, the rocking curve measures the angular distribution (mosaicity) of the VL

orientation in longitudinal direction. For a perfectly straight VL one would expect a sharp peak in the rocking curve at the Bragg angle of the VL, smeared only by the instrument resolution. However, in the presented dataset acquired at 101 mT (Supplementary Figure 1 c) a significantly broadened double peak structure was obtained for both VL Bragg peaks. This is in agreement with the proposed symmetrical bending of the VL within the horizontal plane. The double peak structure in the rocking curve evolves to a single peak in fields above 123 mT. This suggests a straightening of the VL in increasing fields, which can be seen in the change of the TI morphology at this field value as well. It coincides with the IMS to Shubnikov transition. The observed distortion effect is of pure intrinsic nature, as field cooling does not essentially change the rocking curves.

Supplementary Note 2: nGI data processing

Both, the TI and the DFI were processed out of a series of 17 images each taken with an exposure time of 90 seconds. The series of 17 images corresponds to an equidistant stepping of G_0 over one period along the x-direction perpendicular to the grating lines (compare Fig. 4). Thereby the intensity $I(m,n,x)$ in each pixel (m,n) in the detector plane oscillates sinusoidally [41]. To analyse these changes quantitatively, we write the intensity oscillation for each detector pixel in a Fourier series

$$I(m,n,x) = \Sigma_i\, a_i(m,n)\cos(ikx_g+\Phi_1(m,n)) \approx a_0(m,n) + a_1(m,n)\cos(kx_g+\Phi_1(m,n)) \qquad (1)$$

where a_i are the amplitude coefficients, Φ_i the corresponding phase shifts, $k = 2\pi/p_2$, with p_2 being the periodicity of G_2.

The TI of the specimen in each detector pixel is given by

$$TI(m\ n) = a_0\,(\text{sample}) / a_0\,(\text{reference}). \qquad (2)$$

The DFI of the sample in each detector pixel is given by the normalized visibility

$$DFI(m,n) = V(\text{sample}) / V(\text{reference}) \qquad (3)$$

where the visibility $V(m,n)$ is defined by

$$V(m,n) = (I_{max} - I_{min}) / (I_{max} + I_{min}) = a_1 / a_0 \qquad (4).$$

Hence the normalized DFI is given by

$$DFI(m,n) = a_1(\text{sample}) \cdot a_0(\text{reference}) / (a_0(\text{sample}) \cdot a_1\,(\text{reference})) \qquad (5)$$

Simply speaking, the zeroth order Fourier component (offset of the oscillations) provides the TI signal, whereas the first Fourier component (amplitude of the oscillations) provides the DFI signal.

Supplementary Note 3: The sensitivity of the nGI setup

The dark field contrast as defined in Equation 5 describes the local degradation of the nGI interference pattern caused by neutrons been scattered within the sample. In the following we explain how the measured DFI contrast is linked to the samples microstructure and its scattering function *S(q)*. Furthermore, based on simplified considerations we will define the sensitivity of nGI to different sized structures which is necessary to understand the origin of the DFI contrast in the superconducting Nb sample.

Several attempts exist to determine how the nGI interference pattern is influenced by the change of the neutron trajectory within a sample due to scattering. Lynch et al. and Yashiro et al. treated the problem by rigorous first principle wave calculations on different sample to grating configurations [1][2]. They found, that the visibility exponentially decreases with a dark-field extinction coefficient (DFEC) which depends on the autocorrelation function of the refraction index (or the scattering length density) within the material.

A more general and instructive description has recently been given by Strobl [3] which we will summarize in the following. In this model the details of wave propagation are disregarded. Instead, the visibility on the detector decreases due to a superposition of i) the intensity modulation generated by neutrons which pass the sample without scattering and ii) by intensity modulations which are phase shifted due to scattering within the sample. In small angle approximation, the phase shift $\Delta\omega$ of the latter components is directly proportional to the momentum transfer q caused by the scattering event:

$$\Delta\omega = \lambda L_s q/p = \xi_{GI} q, \qquad (6)$$

with the neutron wavelength λ, an effective sample to detector distance L_S and the periodicity of the interference fringes p. The parameter ξ_{GI} hereby defines a correlation length, which is a specific constant for every nGI setup.

For scattering of the whole neutron beam involving only one specific q the visibility would decrease as:

$$DFI = V(sample) / V(reference) = \cos(\xi_{GI} q) \qquad (7)$$

However, if the whole scattering function *S(q)* is taken into account a general solution is derived by integration over all possible q values:

$$DFI = V(sample) / V(reference) = \int_{-\infty}^{\infty} dq S(q) \cos(\xi_{GI} q) = G(\xi_{GI}) \qquad (8).$$

Finally, if multiple scattering is taken into account, a general solution of the DFI contrast is obtained as:

$$DFI(\xi_{GI}) = \exp(\Sigma t\, [G(\xi_{GI}) - 1]). \qquad (9)$$

Therefore, the DFI contrast depends only on the macroscopic scattering cross section of the material Σ, the sample thickness t, and a real space correlation function G. The latter one is the cosine Fourier transformation of the scattering function $S(q)$. It has to be emphasize that despite using different approaches Lynn et al. and Strobl achieve similar results.

Note, that nGI measures a real space correlation at one specific correlation distance ξ_{GI}, instead of a reciprocal space scattering function $S(q)$. Furthermore, these results reveal that there is no direct connection between the structure size and the dark field contrast. Instead, some a priori assumptions about the microstructure (and hence about G) are necessary for a complete modelling of the DFI contrast.

Nonetheless, for the specific case of a diluted systems of identical particles a quantitative definition of the sensitivity to different sized structures is possible. It has been proposed by Lynn et al. to use spherical particles which have a well-known correlation function [4] as reference material. (Note that the term particle-size selectivity is used in [1]). This approach is warranted, as the correlation function $G(r)$ for all diluted systems decays to zero at the longest distance that characterizes the microstructure, which is the diameter in the spherical case. Furthermore the difference in the macroscopic scattering cross section of the particles only multiplies the curve with a constant, leaving its shape unchanged. A more detailed geometrical interpretation of G can be found in [5].

Supplementary Figure 2 shows the calculated DFI contrast of a reference sample consisting a 10 vol% suspension of spherical particles as function of the particle diameter. The contrast was calculated for the used nGI setup parameter based on the formula derived by Lynch [1]. A homogenous sample thickness of 5 mm was assumed. This DFI curve reveals a maximal sensitivity to particles having a size in the range of 1-50 µm, corresponding to the expected size of IMS domains.

The definition of particle-size selectivity via a comparison to microspheres is valid, if no long range order exists in the sample and the systems inter particle distance is large in comparison to ξ_{GI}. We have insofar no evidence that this assumption is not valid within the IMS domain structure as the inter domain distance probed be VSANS seems to be larger than ξ_{GI} and the DFI contrast arises already at 89 mT where a small IMS domain concentration is expected.

In contrast long range order exists within the VL which means that the correlation function has discrete peaks. However, the VL spacing is in the range of 100 nm, thus far away from the probed correlation

length ξ_{GI}~3 µm. Hence, no contribution to the DFI besides the cross talk effect discussed below is expected.

Supplementary Note 4: Crosstalk between TI and DFI

For purely absorbing samples the TI shows contrasts smaller than unity. However, for samples that introduce coherent scattering such as the flux line lattice in superconductors, the TI signal locally shows values larger than unity as shown in Fig. 5 (white lines).

Referring to Eq. 5, the DFI remains smaller than unity as long as the zeroth order Fourier coefficient for the sample is smaller than unity, which is the case if no coherent scattering is present in the sample. However, it is obvious that coherently scattered neutrons locally lead to a larger amplitude a_0(sample). This larger value directly influences the calculated DFI signal as seen in Eq. 5. Under the assumption that the absorption of the sample is negligible, which holds in our case as the data are zero-field normalized, as well as no USANS scattering in the sample is present (a_1 = const.) , the TI and the DFI signals are related by

$$DFI_{Crosstalk} = (a_1(\text{sample}) / a_1(\text{reference}))\cdot(a_0(\text{reference}) / a_0(\text{sample})) = 1 / TI. \qquad (10)$$

According to Eqn. 10 the cross talk of the TI to the DFI can therefore be quantified by $DFI_{Crosstalk} = 1/TI$, namely as the inverted signal of the TI.

In case of the nGI data as presented in Fig. 5 the crosstalk from the TI to the DFI can be better seen by taking horizontal line profiles through the individual TI and DFI images for each magnetic field. These line profiles show that the crosstalk can be directly compared as shown in Supplementary Figure 3.

A true DFI contribution can be seen in the images from 89 mT to 123 mT as the DFI signal shows a stronger contribution than what could arise from the inverted TI crosstalk signal. The data at 112 mT shows the first visible cross-talk between DFI and TI indicated by the two TI peaks which are inverse to the contribution in the DFI at the same horizontal position. At 123 mT the DFI contrast in the center of the sample is solely caused by the TI crosstalk whereas a contribution coming from USANS scattering off the IMS domains is still existing at the sample edge. The DFI signal at 143 mT and 153 mT show the inverse or mirrored signal of what arises from the TI contribution with values larger than unity.

Supplementary Note 5: nGI results obtained in perpendicular beam to field geometry

In this paragraph, results of additional nGI measurements using a perpendicular beam to field geometry are presented.

nGI measurements provide information about the IMS domains and their underlying VL. It has already been shown in the results section that the TI data thereby provide details about the VL structure, whereas the DFI data reveal the distribution of µm VL domains. However, we have shown in the Supplementary Note 4 that a certain amount of crosstalk exists for strong positive TI contrast. This cross talk can be separated from an USANS signal by a comparison of the DFI to 1/TI. Nonetheless, in some cases it is more convenient to choose an alternative experimental configuration in which the field is applied perpendicular to the neutron beam. In this geometry, the VL is rotated out of the Bragg condition preventing SANS scattering which causes the TI signal. In contrast, the isotropic USANS signal, which is mainly caused by the variation of magnetic scattering length density within the VL domain structure, is largely invariant under this rotation and still causes a DFI signal. Hence, by changing the field direction, the TI contrast and consequently the cross talk on the DFI can be eliminated.

To demonstrate this effect, nGI scans of the Nb rod were performed at the ANTARES beamline, operated by TUM at MLZ. The magnetic field was applied in vertical direction along <100> and perpendicular to the neutron beam along <110>. The same sample environment as in former experiments was used. The measurements were performed after ZFC to 4 K. The full neutron spectrum of the white beam was used. The TI and the DFI were processed out of a series of 26 images each taken with an exposure time of 30 seconds. Again, the data in finite magnetic fields are normalized to the data for 0 mT.

The results of the nGI experiment in fields between 75 mT and 143 mT are shown in Supplementary Figure 4. They are again grouped into the TI in the top row and the DFI in the bottom row. As discussed, no magnetic TI contrast arises apart from small contributions due to a slight displacement of the sample holder in higher magnetic fields. However, a pronounced DFI contrast is still visible. Moreover, the field dependence of the DFI contrast variation on the magnetic field is in complete agreement to Fig. 5. Especially at 123 mT, the delayed IMS to Shubnikov phase transition near the cylinder faces is clearly visible as remaining DFI contrast at the left and right edge of the sample.

The presented data further supports the proposed peculiar IMS distribution as other contributions to the DFI contrast than USANS scattering are ruled out. Hence, a perpendicular geometry should always be considered, if one is interested in the domain distribution only and not in the morphology of the underlying VL.

Supplementary References:

[1] Lynch S. K. et al.

Interpretation of dark-field contrast and particle-size selectivity in grating interferometers

Appl. Opt. **50**, 4310 (2011)

[2] Yashiro W., et al.,

On the origin of visibility contrast in x-ray Talbot interferometry

Opt. Express. **18,** 16890 (2010)

[3] Strobl, M.

General solution for quantitative dark-field contrast imaging with grating interferometers

Scientific Reports **4**, 7243 (2014)

[4] Anderson, R. et al.,

Analysis of spin-echo small-angle neutron scattering measurements

J. Appl. Cryst. **41**, 868 (2008)

[5] Krouglov, T. et al.,

Real-space interpretation of spin-echo small-angle neutron scattering

J. Appl. Cryst. **36**, 117 (2003)
